# Deciphering the patterns of genetic admixture and diversity in southern European cattle using genome‐wide SNPs

**DOI:** 10.1111/eva.12770

**Published:** 2019-02-08

**Authors:** Maulik Upadhyay, Chiara Bortoluzzi, Mario Barbato, Paolo Ajmone‐Marsan, Licia Colli, Catarina Ginja, Tad S. Sonstegard, Mirte Bosse, Johannes A. Lenstra, Martien A. M. Groenen, Richard P. M. A. Crooijmans

**Affiliations:** ^1^ Animal Breeding and Genomics Wageningen University & Research Wageningen The Netherlands; ^2^ Department of Animal Breeding and Genetics Swedish University of Agricultural Sciences Uppsala Sweden; ^3^ Department of Animal Science, Food and Nutrition – DIANA, Nutrigenomics and Proteomics Research Centre – PRONUTRIGEN, Biodiversity and Ancient DNA Research Centre – BioDNA Università Cattolica del Sacro Cuore Piacenza Italy; ^4^ CIBIO‐InBIO—Centro de Investigação em Biodiversidade e Recursos Genéticos Universidade do Porto Vairao Portugal; ^5^ Acceligen Recombinetics Saint Paul Minnesota; ^6^ Faculty of Veterinary Medicine Utrecht University Utrecht The Netherlands

**Keywords:** admixture, African taurine, cattle, genetic diversity, haplotype, indicine ancestry, SNPs, southern European

## Abstract

The divergence between indicine cattle (*Bos indicus*) and taurine cattle (*Bos taurus*) is estimated to have occurred approximately 250,000 years ago, but a small number of European cattle breeds still display shared ancestry with indicine cattle. Additionally, following the divergence of African and European taurine, the gene flow between African taurine and southern European cattle has also been proposed. However, the extent to which non‐European cattle ancestry is diffused across southern European cattle has not been investigated thoroughly. Also, in recent times, many local breeds have suffered severe reductions in effective population size. Therefore, in the present study, we investigated the pattern of genetic diversity in various European cattle based on single nucleotide polymorphisms (SNP) identified from whole‐genome sequencing data. Additionally, we also employed unlinked and phased SNP‐based approaches on high‐density SNP array data to characterize non‐European cattle ancestry in several southern European cattle breeds. Using heterozygosity‐based parameters, we concluded that, on average, nucleotide diversity is greater in southern European cattle than western European (British and commercial) cattle. However, an abundance of long runs of homozygosity (ROH) and the pattern of Linkage disequilibrium decay suggested recent bottlenecks in Maltese and Romagnola. High nucleotide diversity outside ROH indicated a highly diverse founder population for southern European and African taurine. We also show that Iberian cattle display shared ancestry with African cattle. Furthermore, we show that Podolica is an ancient cross‐bred between Indicine zebu and European taurine. Additionally, we also inferred similar ancestry profile of non‐European cattle ancestry in different Balkan and Italian cattle breeds which might be an indication of the common origin of indicine ancestry in these breeds. Finally, we discuss several plausible demographic scenarios which might account for the presence of non‐European cattle ancestry in these cattle breeds.

## 1 INTRODUCTION

Modern cattle originated from at least two different species of wild aurochs: *Bos primigenius primigenius* (European aurochs) and *Bos primigenius namadicus* (Indian aurochs). Analyses based on mitochondrial DNA (mtDNA) have estimated the divergence date between these two species from ~117,000 to ~332,400 years before present (YBP) (Achilli et al., 2008; Bradley, Machugh, Cunningham, & Loftus, 1996; Loftus, Machugh, Bradley, & Sharp, 1994). Subsequently, the near‐Eastern population of *Bos. p. primigenius* was domesticated ~10,000 YBP giving rise to domesticated taurine population (Troy et al., 2001), while *Bos. p. namadicus* was domesticated ~8,000 YBP, somewhere in the Indus Valley giving rise to the modern zebu (cattle with hump aka indicine) population (Chen et al., 2010). The occurrence of a third domestication event involving African aurochs in north‐eastern Africa is still debated (Grigson, 1991; Loftus et al., 1994; Pitt et al., 2018). However, recent studies based on genome‐wide single nucleotide polymorphisms (SNPs) refuted this hypothesis of a third domestication centre and, instead, proposed the gene flow from African aurochs resulting in high divergence of African taurine (Decker et al., 2014; Pitt et al., 2018). This hypothesis of African aurochs introgression, however, is yet to be tested because DNA from an ancient specimen of African cattle is not available. Conversely, analyses of genome‐wide SNPs from ancient European aurochs have provided novel insights into the post‐domestication contribution of ancestral aurochs to the gene pool of modern European cattle. For instance, studies have suggested that British cattle followed by Iberian and Dutch cattle may carry an abundance of aurochs specific alleles compared to either African or near‐Eastern taurine breeds (Park et al., 2015; Upadhyay et al., 2016). More aurochs samples from different parts of Europe and at different time points, however, are needed for further validation of these results.

Based on the archaeological evidences (Martins et al., 2015; Zilhao, 1993) and genomic analysis of DNA sequences of ancient farmers (Hofmanová et al., 2016), at least two migration routes have been suggested to explain the expansion of Neolithic farmers along with their domesticated animals after early domestication of European aurochs in the Near East. Following these early migrations, various instances of gene flow involving non‐European cattle into the gene pool of southern European cattle have been suggested. For instance, mtDNA, microsatellite markers, and genome‐wide SNP‐based studies have shown the presence of African taurine ancestry in Iberian breeds (Beja‐Pereira et al., 2003; Cymbron, Freeman, Isabel Malheiro, Vigne, & Bradley, 2005; Decker et al., 2014; Ginja, Penedo et al., 2010; Ginja, Telo Da Gama, & Penedo, 2010). On the other hand, several Italian breeds also carry complex non‐taurine ancestry. For instance, analysing microsatellite markers, Cymbron et al. (2005) reported the highest frequency of indicine population‐associated alleles in Greek and Italian cattle breeds, while Decker et al. (2014) reported the dual ancestry—African taurine as well as indicine—in three Italian cattle breeds, namely Chianina, Romagnola and Marchigiana. However, the origin of such dual ancestry remains unclear. Moreover, it is also not known whether other Balkan and Italian (BAI) cattle breeds, such as Busha and Maremmana, also carry the similar dual ancestry. Further, the genetic admixture pattern in southern European cattle has mostly been investigated using either mitochondrial DNA (Di Lorenzo et al., 2018; Pellecchia et al., 2007) or microsatellite markers (D'Andrea et al., 2011). Conversely, genome‐wide high‐density SNP markers have scarcely been used for detailed characterization of the non‐European ancestry in southern European cattle.

The European indigenous native cattle breeds are valuable genetic resources as they are well adapted to local environments. For instance, Maremmana became well adapted to the hot and humid environment of Tuscan Maremma plain, once wetlands where malaria was endemic. Further, it has been postulated that some local breeds like Busha might have conserved an abundance of rare alleles because of their large effective population sizes (Medugorac et al., 2009). Indeed, conservation analyses have prioritized some local European cattle for conservation, namely from Iberia (Canon et al., 2001; Ginja et al., 2013). Additionally, in some cases, the certified products obtained from local breeds provide an additional value that distinguishes them from non‐native breeds (Di Trana et al., 2015). Furthermore, local breeds are also attached to several traditions of cultural heritage. The rapid decline of local breeds, however, remains a major concern. It has been estimated that of the 640 global cattle breeds with known “risk status,” 171 breeds can be classified under “at risk” category while 184 breeds are already extinct (FAO, 2015). In our previous study, we also reported the recent reduction in effective population size for several southern Europe breeds such as Mirandesa and Maltese (Upadhyay et al., 2016). Therefore, a comprehensive understanding of the status of current genetic diversity and demographic processes driving these changes will have a large impact on their ongoing conservation efforts.

In this study, genome‐wide SNP data were generated using two different approaches: BovineHD SNP array genotyping and whole‐genome sequencing (WGS). At first, we used the genotypes obtained from WGS of European cattle to assess genetic heterozygosity and change in recent demography, followed by identification and assessment of non‐European ancestry in southern European cattle (Iberian, and BAI cattle) using BovineHD SNP array data.

## 2 MATERIALS AND METHODS

### 2.1 Whole‐genome sequencing data, alignment and variant calling

Blood, hair roots or semen samples were collected from twelve individuals from BAI cattle breeds (one Boskarin, two Busha, one Chianina, four Maltese, one Italian Podolica and three Maremmana) and seven individuals from Iberian cattle breeds (one Limia, one Maronesa, two Pajuna, two Sayaguesa and one Tudanca). DNA was extracted either using QIAamp DNA blood spin kit (Qiagen Sciences) or DNeasy^®^ Blood & Tissue Kit (Qiagen Sciences). DNA quantification and qualification were carried out using Qubit 2.0 fluorometer (Invitrogen). Library construction was carried out with 0.5–3 µg of genomic DNA following the Illumina library prepping protocols (Illumina Inc.).

All the 19 individuals were paired‐end re‐sequenced with the Illumina sequencing technology (Illumina Inc.). We also obtained additional 18 WGS data (15 raw sequenced data and three WGS alignment) of several commercial and traditional cattle from previous studies (Bickhart et al., 2016; Daetwyler et al., 2014; Kim et al., 2017; Murgiano et al., 2014). All the detailed sample information is given in Supporting Information Table S1. To perform the quality‐based trimming on each fastq file, sickle (Joshi and Fass et al., 2011) was run with default settings except for the length threshold of 50 bp. Following this trimming, BWA‐mem (Li, 2013) algorithm was used to align the quality‐trimmed fastq files against the bovine reference genome build UMD 3.1. After the alignment, duplicate reads were removed from the bam files using “Samtools rmdup” (Li et al., 2009). Finally, “RealignTargetCreator” and “IndelRealigner” arguments as implemented in Genome analysis toolkit 3.1 (GATK) (Li et al., 2009) were used to perform local read realignments.

Multi‐sample variant calling was carried out using freebayes (Garrison & Marth, 2012) with the default settings except for the parameters minimum base quality (‐‐min‐base‐quality 20) and haplotype length (‐‐haplotype‐length 0). Under the default parameters, freebayes only considers the alternate allele as a SNP if it is covered by at least two reads or present in at least 0.2 fractions of the total reads aligned at a position in at least one sample. Following this early round of SNP calling, vcftools (Danecek et al., 2011) was used to discard variants using these criteria: (a) indel positions, (b) SNP variants with more than two alleles (c) SNP variants with genotyping quality less than thirty and (d) SNP with a minimum depth less than four and a maximum depth greater than thirty (avoid erroneous genotypes due to CNV). The concordance between genotypes as called from the whole‐genome sequencing data set and BovineHD SNP array data set was examined using seven samples for which both genotype sets were available.

### 2.2 Estimation of heterozygosity using whole‐genome sequences

The genetic heterozygosity was estimated in each individual whole‐genome sequence using mlRho (Haubold, Pfaffelhuber, & Lynach, 2010). The method implemented in mlRho co‐estimates the population mutation rate (Ɵ) and sequencing error rate (є) using maximum‐likelihood approach. If the value of Ɵ is small, the estimate of theta approximately reflects heterozygosity under an infinite allele model. For this analysis, we used UMD 3.1 aligned data with mapping quality >15, base quality >25 and sites with the depth between 4 and 30.

### 2.3 Assessment of recent demographic change using runs of homozygosity (ROH) analysis

Runs of homozygosity (ROHs) were extracted from whole‐genome sequence data following the procedure implemented as in Bosse et al. (2012), using sliding windows approach of 10 kbps. We defined an ROH as a genomic region of at least 20 kbps where the number of heterozygous SNPs per bin (or SNP count) was less than 0.25 times the whole‐genome nucleotide diversity, and at least 20 consecutive bins showed a total SNP average lower than the total genomic average. Local assembly or alignment errors were minimized by relaxing the threshold for individual bins within a candidate homozygous stretch, allowing the number of SNPs per bin to be maximum twice the genomic average and the average SNP count within the candidate ROH to not exceed 2/3 the genomic average. Both whole‐genome nucleotide diversity and nucleotide diversity within a candidate ROH were estimated from well‐covered sites only, which were defined by a depth of coverage between 4× and 30×.

### 2.4 BovineHD genotyping array data and filtering

BovineHD SNP genotyping array data of various European, African and Indian cattle as published in the previous studies (Bahbahani, Tijjani, Mukasa, & Wragg, 2017; Barbato et al., unpublished; Upadhyay et al., 2016) were obtained and merged for the top alleles using PLINK v1.07 (Purcell et al., 2007). For the present study, several additional animals of various Italian and Iberian cattle breeds were genotyped using BovineHD SNP array. The merged BovineHD genotyping array data were filtered using PLINK (Purcell et al., 2007) to keep the animals with more than 90% of genotypes called (‐‐mind 0.1), SNPs that were present across at least 95% of the samples (‐‐geno 0.05), and SNP with minor allele frequency ≥1% (‐‐maf 0.01). The complete total BovineHD genotyping data set consisted of about 670 k SNPs and 358 samples (Supporting Information Table S2).

### 2.5 Population admixture using the unlinked SNPs

Genetic admixture patterns of southern European cattle breeds in relation to non‐European taurine and zebu were characterized using (a) model‐based clustering as implemented in ADMIXTURE (Alexander, Novembre, & Lange, 2009), (b) treemix analysis (Pickrell & Pritchard, 2012) and (c) three‐population test (Reich, Thangaraj, Patterson, Price, & Singh, 2009). The ADMIXTURE analysis was carried out with 1,000 bootstrap replicates for population cluster (*K*) values from 2 to 6. Prior to the ADMIXTURE analysis, a linkage disequilibrium (LD) pruning was performed, to reduce the overall pairwise LD to <0.10. We used the python package *pong *to generate the figures based on ADMIXURE result (Behr, Liu, Liu‐Fang, Nakka, & Ramachandran, 2016). Treemix analysis (Pickrell & Pritchard, 2012) was carried out to investigate the migration events of zebu and African cattle during the domestication history of European taurine. In brief, we followed the procedure of Decker et al. (2014): first, we generated maximum‐likelihood‐based phylogenetic tree of all cattle populations, and iteratively, we added one migration edge to the previously generated graph with “m” migration edge. We rooted the graphs with yak (as an outgroup), used blocks of 1,000 SNPs and applied the “‐se” option to estimate standard errors of migration proportions. For this analysis, the scaffolds of yak genome assembly (Hu et al., 2012; Qiu et al., 2012) were aligned to the bovine UMD 3.1 assembly and processed as described in Upadhyay et al. (2016). Moreover, the SNP genotyping data of the British aurochs (Park et al., 2015) were also used in this treemix analysis. The treemix analysis was run five separate times, each time using different seeds, to assess the consistency of migration edges across different runs. Additionally, the f3 tests, which consider the correlation of allele frequencies across the genome‐wide markers, were also carried out to provide support to admixture analysis. The f3 tests with *Z*‐score less than −3.0 were considered as significant. The three‐population tests were carried out using ADMIXTOOLS version 4.1 (Patterson et al., 2012).

### 2.6 Population admixture using the phased SNPs

#### 2.6.1 Phasing

We phased genotypes of each autosomal chromosome separately using Beagle 4.1 (Browning & Browning, 2007) by setting all the parameters as default. The recombination map of cattle was used from the previous study (Ma et al., 2015). This recombination map comprises of 59,309 SNP markers for 29 autosomes with an average distance of 0.043 cM in males and 0.039 cM in females. Our data, however, consisted of ~670 K SNPs, and hence, for an SNP with unknown recombination rate in our data set, we assign it the value of nearest SNP with a known location in recombination map, thus keeping the overall genetic distance between markers same as that of the consecutive markers in original recombination map (Ma et al., 2015).

#### 2.6.2 ChromoPainterv2 to infer the coancestry matrix

To infer population admixture using the phased data, Li and Stephens (2003) algorithm as implemented in ChromoPainterv2 (Lawson, Hellenthal, Myers, & Falush, 2012) was used. The underlying algorithm takes into consideration LD and underlying recombination process along the markers and reconstructs each haplotype as a recipient of a series of genetic chunks from all the other “donor” haplotypes. ChromoPainterv2 first calculates nuisance parameters, *n* (like effective population size) and *M* (population mutation rate), to implement them in a probabilistic model that calculates copying probability of genetic chunks in a recipient haplotype given the other donor haplotypes. We used SNPs located on chromosomes 1, 2, 7 and 12 to infer the “n” and “M” using 10 iterations of expectation maximization (EM) algorithms. The obtained values of “n” and “M” were 307 and 0.0012, respectively. Finally, these inferred values were fixed in the algorithm to obtain the ChromoPainter coancestry matrix (count matrix as well as length matrix) that measures the haplotype sharing among the samples across all the chromosomes.

#### 2.6.3 FineSTRUCTURE algorithm to cluster the samples

Once we obtained the count of shared haplotypes between different samples, we used the FineSTRUCTURE to cluster the samples into genetically homogeneous groups. Following Leslie et al. (2015), FineSTRUCTURE was run for 2 million Markov chain Monte Carlo (MCMC) iterations with the initial 1 million iterations discarded as “burn‐in”, and following these “burn‐in,” sampling from the posterior distribution was carried out at every 10,000 iterations. Later, as recommended in Lawson et al. (2012), we ran 10,000 “hill climbing” iterations on the MCMC iteration with the highest posterior probability to get the final cluster assignment.

#### 2.6.4 GLOBETROTTER to estimate the admixture proportion

The multiple linear regression model as implemented in the GLOBETROTTER algorithm was used to assess the ancestral make‐up of BAI cattle breeds in terms of ancestry contribution from various taurine and zebu clusters. In brief, we model the genome of each BAI cattle breed as a linear mixture of the taurine and zebu donor populations using the method described in Leslie et al. (2015). To estimate the standard error in these ancestry proportions, we applied delete‐one chromosome jackknife approach (Busing, Meijer, & Leeden, 1999) as described in Montinaro et al. (2015).

As the estimates of admixture dates and ancestry proportions depend on the true underlying recombination rate between any two consecutive SNPs, we interpolated true average recombination rate based on the cattle recombination map constructed by Ma et al. (2015).

### 2.7 Linkage disequilibrium decay and estimation of effective population size

To assess the change in effective population size and the pattern of LD decay, the SNeP v1.11 (Barbato, Orozco‐terWengel, Tapio, & Bruford, 2019) software was used. The LD decay analysis was run with the following parameters: ‐mindist 5,000, ‐maxdist 2,000,000, ‐numBINS 50, ‐maf 0.05.

## 3 RESULTS

### 3.1 Profile of heterozygosity and runs of homozygosity

Individual genomes of 19 southern Eastern European cattle were sequenced and genotyped along with 18 additional whole‐genome sequences downloaded from NCBI SRA (detailed information in Supporting Information Table S1). These additional sequences were comprised of individuals from Indian zebu (two Gir), African zebu (two Kenana), African taurine (four N'Dama), British (two Belted Galloway, two Dexter, one Hereford), Italian (two Romagnola) and commercial (one HF, Jersey and Simmental each) cattle breeds. This sampling scheme allowed to compare the genetic diversity and recent demographic changes between cattle from a wide range of geographical regions. The coverage of re‐sequenced genomes across samples in the final bam files varied from ~2.6× to ~16× (Supporting Information Table S1). The alignment was performed against UMD3.1 using bwa‐mem, and the alignment rate was greater than 98% for all the samples. The total number of SNPs identified after performing quality filtering in bam file and post‐SNP calling was greater than ~30 million. The genotyping concordance (Supporting Information Table S1) between SNPs genotyped using the BovineHD SNP array and SNPs genotyped using whole‐genome sequencing was approximately 94% for all seven samples, probably indicating the low proportion of false positive SNPs in the data set.

**Figure 1:**
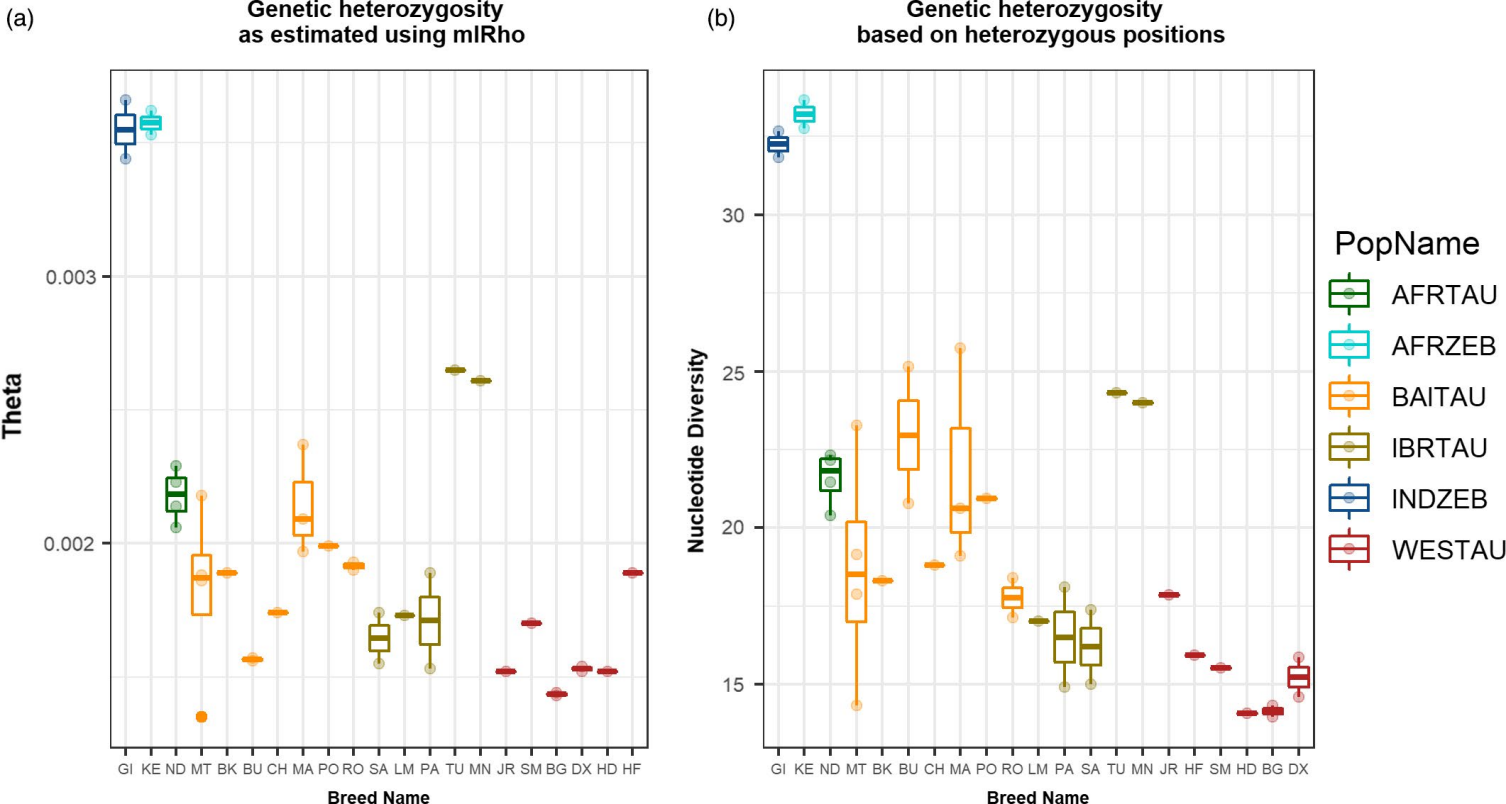
Boxplots showing average population scaled mutation rate (a) and average heterozygosity calculated in 10‐kbp window using whole‐genome sequencing data. AFRTAU: African Taurine; AFRZEB: African Zebu; BAITAU: Balkan and Italian Taurine; IBRTAU: Iberian Taurine; INDZEB: Indian Zebu; WESTAU: western European Taurine. Refer to Supporting Information Table S1 for the breed abbreviations

We used two different approaches to estimate heterozygosity from whole‐genome sequences of cattle. Both the approaches—population mutation rate (Ɵ) and nucleotide diversity calculated as an average number of heterozygous sites in a 10‐kbp window—indicated relatively low heterozygosity in western European cattle, while several southern European individuals displayed the levels of heterozygosity comparable to African taurine (Figure 1). All Maremmana (MA) individuals consistently displayed a high value of heterozygosity in both the approaches (Figure 1a,b). The African zebu cattle displayed the highest values of heterozygosity, probably due to the admixed nature of their genome (Bahbahani et al., 2017).

**Figure 2:**
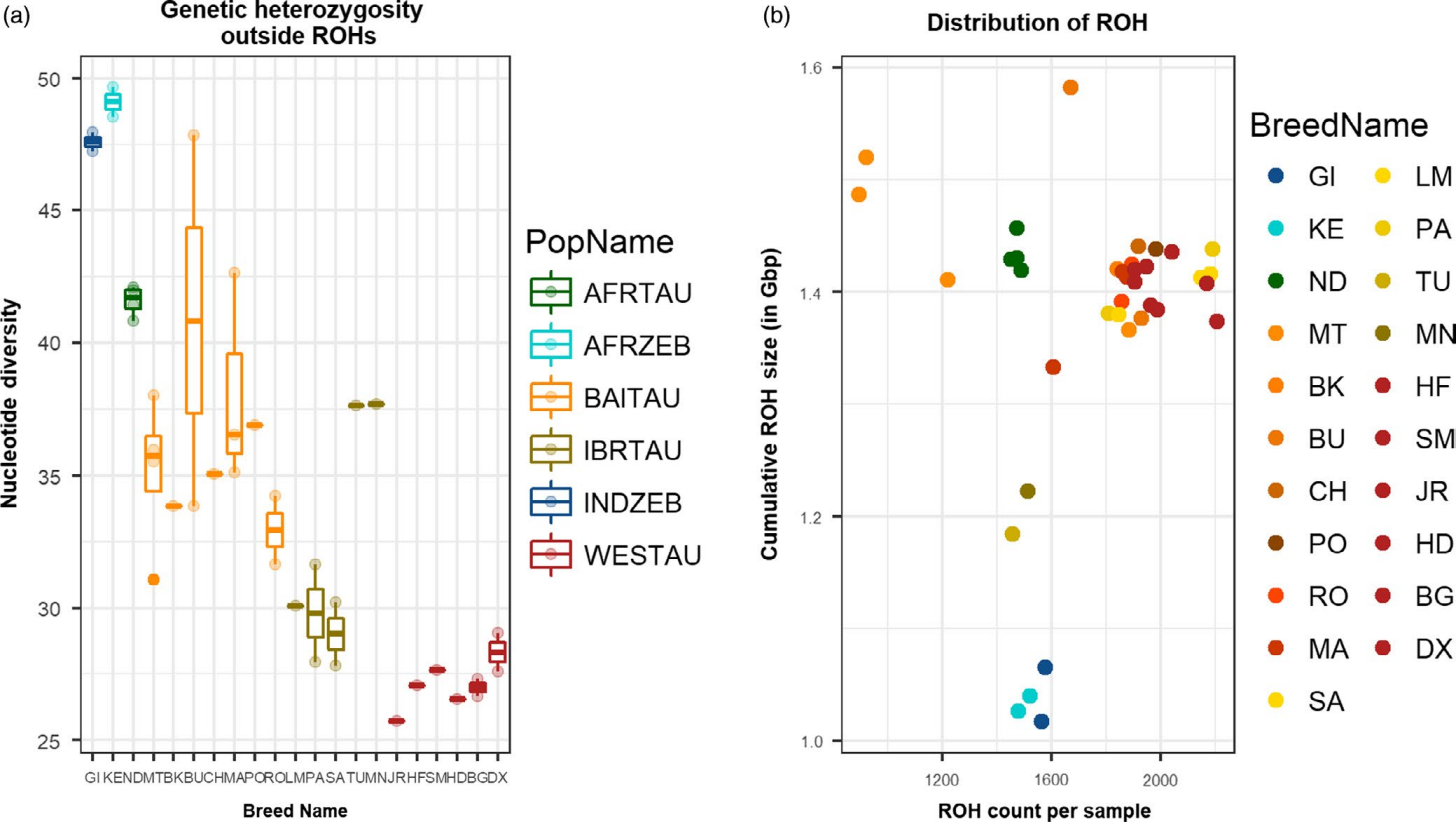
Boxplots showing average heterozygosity calculated in 10‐kbp window outside ROH (a) and distribution of ROH (b) using whole‐genome sequencing data. AFRTAU: African Taurine; AFRZEB: African Zebu; BAITAU: Balkan and Italian Taurine; IBRTAU: Iberian Taurine; INDZEB: Indian Zebu; WESTAU: western European Taurine. Refer to Supporting Information Table S1 for the breed abbreviations

The number and total length of ROHs in the genome did not vary sharply among cattle from different regions (Figure 2b). In concordance with the previously described heterozygosity estimates, several southern European individuals displayed the number and cumulative length of ROHs comparable to that of N'Dama cattle. On an average, Maltese as well as N'Dama individuals, despite the low number of ROHs in their genome, has a substantial part of their genome under ROHs, indicating a significant contribution from long ROHs. This is an indication of a recent reduction in the effective population size of Maltese cattle. The commercial and British individuals displayed the highest number of ROHs as well as a high cumulative length of ROH (Figure 2b), probably a result of intense selection. We also estimated the nucleotide diversity outside the ROH regions (π_out), and the result (Figure 2a) consistently indicated greater values in southern European individuals compared to the west European cattle. Also, N'Dama individuals have the highest π_out values among all the taurine cattle. These results could indicate that southern European and African taurine cattle are descendant of diverse ancestral population. We note that the ROH profile (Figure 2b) of Busha, which is characterized by the low number and low cumulative length—might also be a consequence of their low genome coverage (~2–4×).

### 3.2 Investigation of genetic admixture using unlinked SNPs

**Figure 3:**
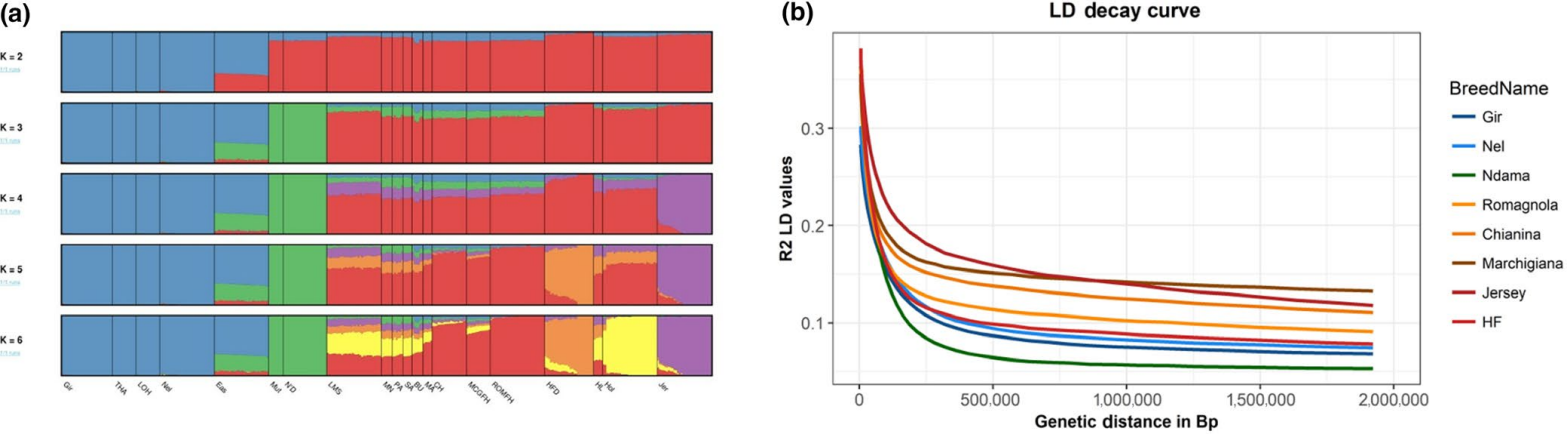
Unsupervised hierarchical clustering (a) showing results for an inferred number of clusters, *K* varying from 2 to 6. Linkage disequilibrium decay analysis (b) comparing central Italian cattle breeds against commercial cattle breeds. Refer to Supporting Information Table S2 for the breed abbreviations

To investigate the presence of indicine and African cattle ancestry among southern European cattle, admixture analysis was initially run on the entire data set of 358 individuals for values of *K* between 2 and 11. For values of *K* from 2 to 6, all Iberian samples display minor component of African cattle ancestry in their genome (Figure 3a). Among BAI breeds, Busha (BU), Maremmana (MA), Chianina (CH) and Marchigiana (MCGFH) display African as well as indicine cattle ancestry in their genome. On the other hand, at the value of *K* = 5 and *K* = 6, Romagnola (ROMFH) displays only single ancestry which is also one of the ancestral components in almost all European taurine samples. We hypothesized that this unique cluster of Romagnola is the result of a significant bottleneck due to a recent reduction in the effective population size. To test this hypothesis, we performed linkage disequilibrium decay analysis to estimate the change in effective population size for all the breeds with sample size more than ≥13 (Figure 3b). The analysis indicated greater historical effective population size, but slow LD decay in Italian cattle breeds compared to the commercial cattle breed HF. Such slow LD decay indicates the presence of long haplotypes which are usually consequences of bottleneck event.

**Figure 4:**
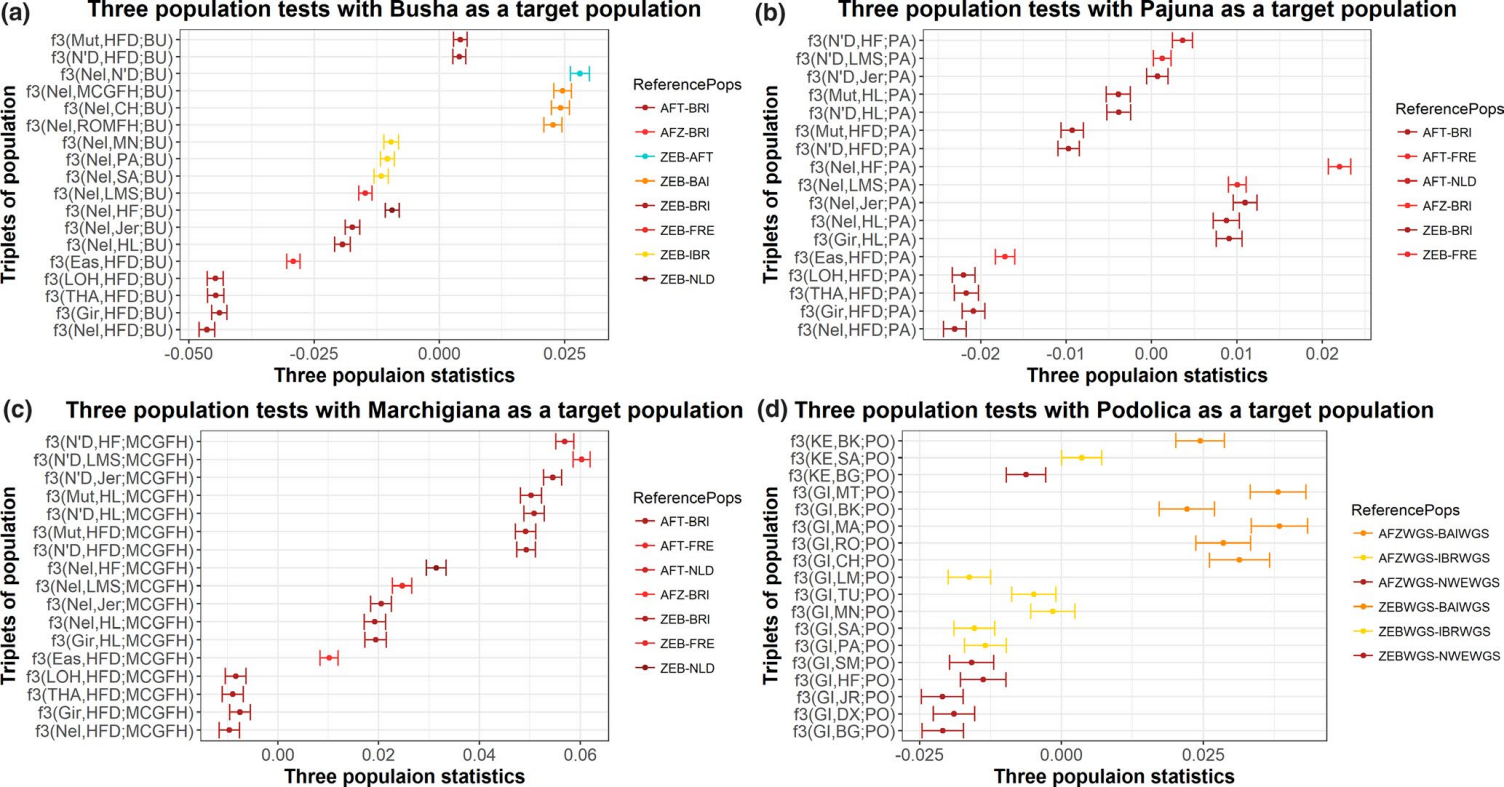
Three‐population tests with Busha (a), Pajuna (b), Marchigiana (c) and Podolica (d) as target populations. In the case of Podolica, f3 tests were performed on whole‐genome sequencing data, while for the remaining population, f3 tests were performed on SNP array data. The dot shows f3 statistics value, while the horizontal bar shows a plus or minus standard error. Refer to Supporting Information Table S2 for the breed abbreviations

To supplement the admixture analysis, the f3 tests (Figure 4) were also carried out to determine whether any southern European cattle breed could be modelled as an admixture between European taurine and other non‐European cattle. The f3 tests, which were performed with British, Dutch or Iberian breeds as one of the reference population and any zebu breeds as another reference population, resulted in significant Z‐scores for the target population of Busha (Figure 4a). This provided strong evidence in favour of admixed origin of Busha. In case of Pajuna, only the tests with Hereford (HFD) as one of the reference population and any African taurine or any zebu cattle as another reference population resulted in significant negative Z‐scores (Figure 4b). A similar pattern (Figure 4c) was observed in Marchigiana as well; all the f3 tests involving Hereford (HFD) and any of the zebu cattle breeds as reference populations resulted in significant Z‐scores. These results could indicate that British cattle are the most suitable surrogates (in our data) for the European taurine that contributed to Busha, Pajuna and Marchigiana cattle ancestry or it could also indicate an artefact of the ascertainment bias of BovineHD SNP array. Though Patterson et al. (2012) have shown that, on the whole, f3 tests are unaffected by SNP ascertainment bias, they also indicated that differential population frequency, just by chance, could also lead to false positive signals. To test this hypothesis, we performed three‐population tests using two sets of genotypes obtained from WGS data: (a) genotypes identified from aligning the cattle sequences against the taurine reference (UMD 3.1) and (b) genotypes identified from aligning the cattle sequences against indicine reference (Bos_indicus_1.0). Interestingly, across both genotype sets, only a Podolica sample generated significant negative Z‐scores for f3 tests with British, Dutch or Iberian cattle as one and Gir as another reference population (Figure 4d).

**Figure 5:**
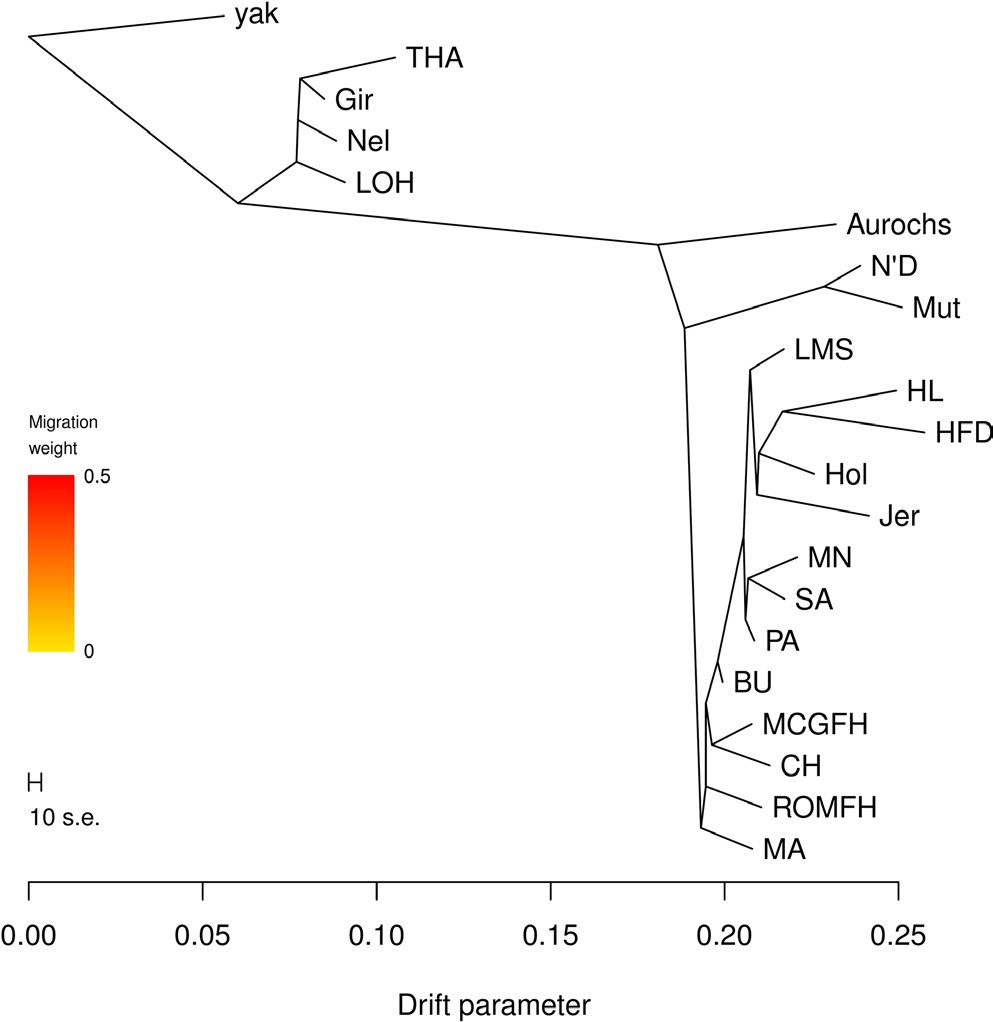
Maximum‐likelihood‐based phylogenetic tree. Scale bar shows 10 times the average standard error of the estimated entries in the sample covariance matrix. Refer to Supporting Information Table S2 for the breed abbreviations

To investigate the cattle phylogeny and relationship across Indicine, African and taurine lineages, the phylogenetic tree was constructed by applying the maximum‐likelihood (ML) algorithm as implemented in the software treemix. The ML‐based phylogenetic tree (Figure 5) topology perfectly captures known relationships among taurine and zebu cattle populations. Using Yak as an outgroup, the tree displays the first split between taurine and zebu cattle which is followed by a split between domestic taurine and wild British aurochs. African taurine appears to be the most divergent among all domestic taurine, while BAI breeds appear to be paraphyletic. All Iberian breeds display short branch lengths and form a single clade.

To investigate the migration events involving indicine and African cattle in southern European cattle, we ran five independent treemix analysis, each initialized with different random seeds. Across all the runs, the graph model without any migration edges explained about 98.73% of the total variance (Supporting Information Figure S1) in relatedness between populations meaning that adding migration events would improve the fit within a graph. Across all the treemix runs (Supporting Information Figure S2), we either observed migration edges between African taurine and Iberian breeds, or the same clade for African taurine and Iberian samples. Interestingly, all BAI breeds displayed inconsistent migration edges across all the different treemix runs (Supporting Information Figure S2). Decker et al. (2014) also reported inconsistent placement of Italian breeds across the different treemix runs, probably as the result of an underlying complex phylogeny.

### 3.3 Investigation of admixture pattern using phased SNPs

**Figure 6:**
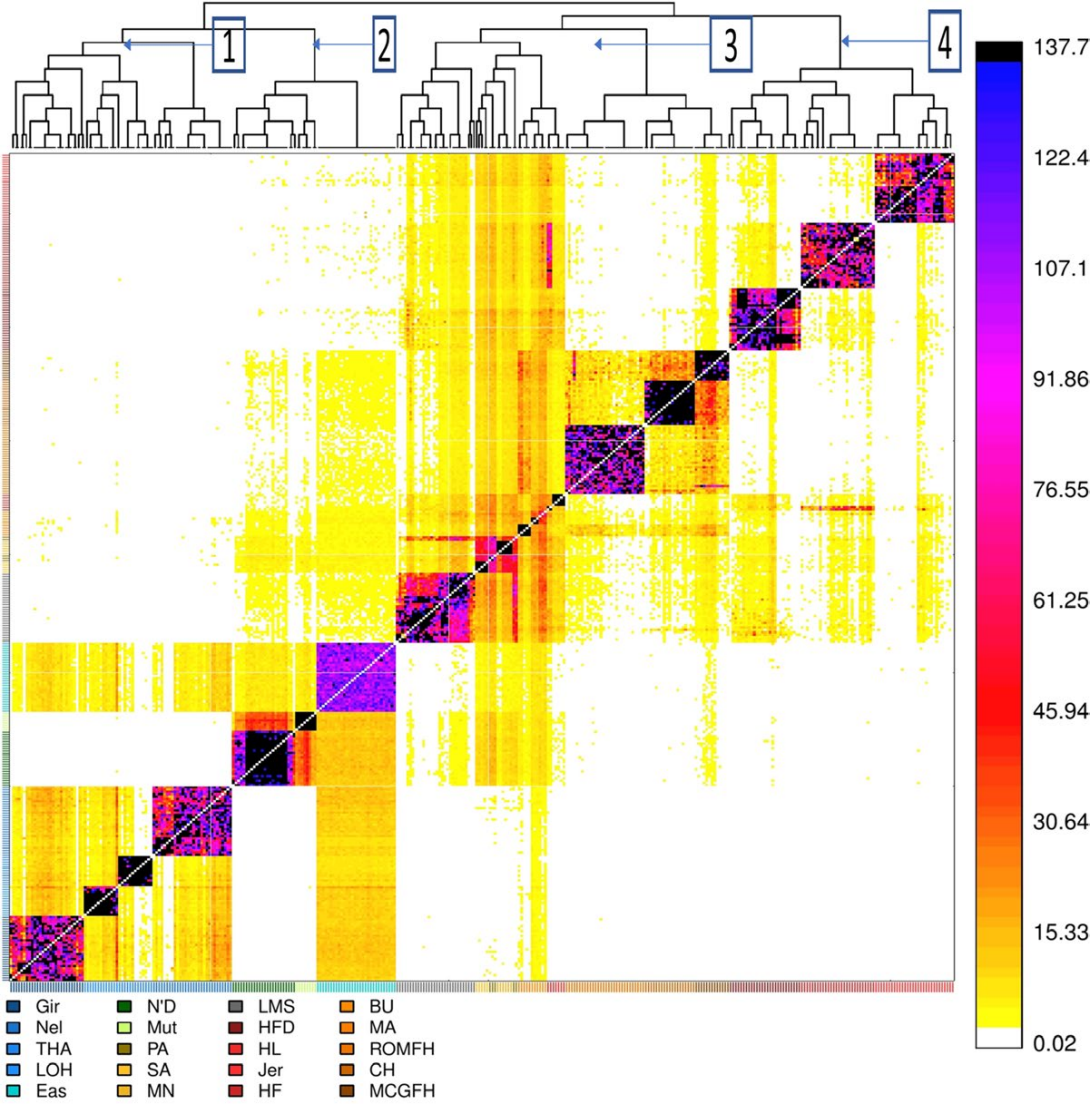
Clustering of individuals based on fineStructure algorithm. Note that breeds are coloured according to their geographic origin. The intensity of colour indicates shared haplotypic segments (values in terms of centiMorgan) based on chunklength coancestry matrix generated by ChromoPainter algorithm. The number beside the clusters indicates (1) Zebu cattle, (2) African cattle (N'Dama and EAZ), (3) southern European cattle (Iberian and Italian) and (4) west European cattle (commercial and British cattle). Note that African cattle clusters with European when performed the same analysis but with a smaller number of zebu and taurine samples (Supporting Information Figure S3). Refer to Supporting Information Table S2 for the breed abbreviations

The pattern of haplotype sharing and in‐depth analysis of the complex admixture pattern of southern European cattle were investigated by ChromoPainter and fineSTRUCTURE. The pattern that emerged from ChromoPainter coancestry matrix (Figure 6 and Supporting Information Figure S4) as well as the clustering of samples based on the fineStructure analysis not only reinforced the finding of the ADMIXTURE and treemix analysis but also provided additional insight into the relationships between zebu and taurine cattle. Based on the fineSTRUCTURE‐inferred tree, we classified our cattle data set of 358 animals into the following major clusters: (a) indicine zebu, (b) African cattle, (c) southern European cattle and (d) western European cattle. In indicine zebu cluster, we observe that Nellore (NEL) forms a separate subcluster from Gir, Tharparkar (THA) and Lohani (LOH) which could be attributed to the fact that Nellore is derived from south‐eastern Indian zebu, while the remaining are from north‐western Indian subcontinent. In African cattle cluster, we observed that N'Dama (N'D) and East African Zebu (EAZ) display a significant haplotype sharing which is consistent with the hybrid history of EAZ. It is worthwhile to note that EAZ also displays significant haplotype sharing with southern European cattle. Among the southern European cattle, Limousin (LMS) forms a cluster with Iberian cattle (Pajuna (PA), Sayaguesa (SA) and Maronesa (MN)) and they both display a significant haplotype sharing with African taurine and EAZ. The haplotype sharing pattern of Maremmana (MA) and Busha (BU) represents the mixture of a genetic component of all the cattle present in the data set which can be attributed to their small sample size. Nevertheless, ADMIXTURE analysis also represented the Maremmana (MA) and Busha (BU) ancestry as a mosaic of all genetic ancestry of all the cattle in the data set. However, among the Italian cattle breeds, only Marchigiana (MCGFH) recorded significant length of haplotype sharing with non‐European cattle, which could be due to differences in genetic drift after splitting from the common ancestor.

**Figure 7:**
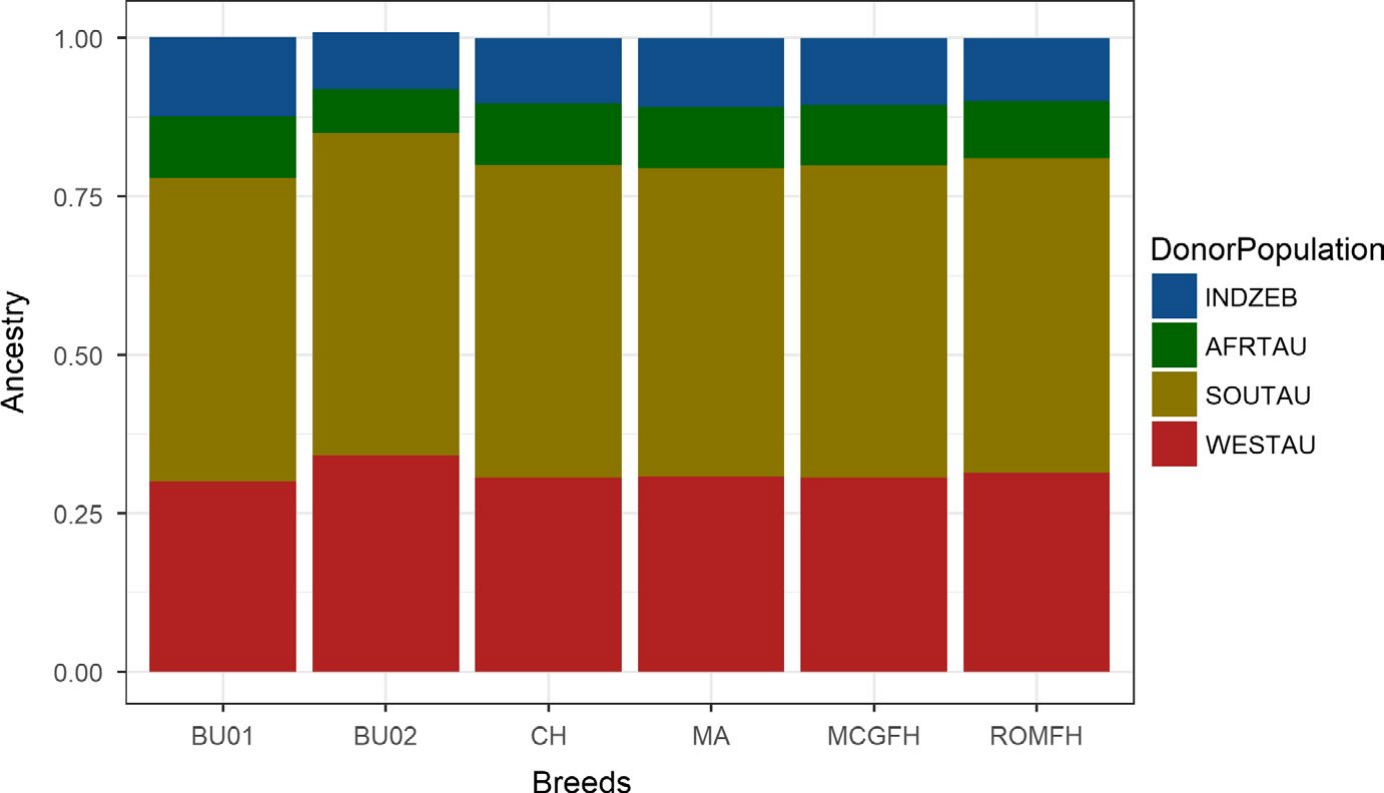
Ancestry proportion of different donor populations in the genome of Italian and Balkan cattle breeds. INDZEB: Indian Zebu; AFRTAU: African taurine; SOUTAU: southern European taurine (without Italian cattle breeds); WESTAU: western European taurine. Note that Busha individuals come from two different subpopulations and hence treated separately in this analysis. Refer to Supporting Information Table S2 for the breed abbreviations

To account for the drift effect, we re‐run the ChromoPainter analysis with Italian breeds only allowed to receive the ancestry from the non‐Italian cattle donor groups (four clusters) assigned based on the fineStructure‐inferred tree. Also, we did not consider EAZ as donor population because it is the cross‐bred of African taurine and indicine zebu. With this analysis, we specifically wanted to compare the ancestry profile of different BAI breeds. In particular, we note that if introgression events involving non‐European cattle ancestry are ancient and split between Italian cattle is relatively recent, then all Italian cattle would display similar ancestry profile. The ancestry profile of Italian cattle displays similar zebu and African taurine ancestry (Figure 7). The zebu and African taurine ancestry (Supporting Information Table S3) in these cattle breeds varied between approximately 9%–12.5% (except BU02) and 8%–10%, respectively.

## 4 DISCUSSION

### 4.1 Genetic diversity and change in recent demography

The average autosomal heterozygosity estimated using mlRho did not sharply contrast the cattle from different regions except that, on average, zebu and African cattle showed greater heterozygosity compared to the European cattle (Figure 1). However, differences in the estimates between different breeds were clearly visible. For instance, all Maremmana (MA), Romagnola (RO) and Podolica (PO) individuals showed greater heterozygosity compared to all western European cattle. In fact, all the estimates for Maremmana (MA) were either equal to or more than the previously reported estimates for European commercial European cattle that ranged from 1.27 × 10^−3^ to 1.97 × 10^−3^ (Gautier et al., 2016). As intensive artificial selection or/and genetic isolation usually led to a reduction in effective population size and associated genetic diversity, the low values for the estimates were expected for western European cattle. On the other hand, high genetic diversity in Maremmana (MA), Podolica (PO) and Romagnola (RO) can be attributed to their relatively shorter history of artificial selection and greater historical effective population size compared to western European (WESTAU) cattle (Figures 1 and 2). However, we also note that LD decay analysis using SNP array markers indicates a recent effective population size in Romagnola (ROMFH) that is comparable to that of the commercial cattle (Figure 2). This result can be attributed to the strong decline in the effective population size of Romagnola.

The fact that Maltese (MT) displayed large between the individual variations in theta as well as in ROH profile indicates that genetic substructure exists in a population (Figure 2). This genetic substructure may have been formed as a result of the varying degree of Chianina admixture in a population (Lancioni et al., 2016). Also, Maltese (MT) individuals display a large proportion of genome under long ROHs indicating a strong recent bottleneck event (Figure 2b). This result is in good agreement with our previous study, where we observed a similar long ROH pattern using BovineHD SNP array (Upadhyay et al., 2016).

The nucleotide diversity outside ROH is a good indicator of ancestral haplotype diversity as it reflects the haplotype variations that remained weakly affected by recent consanguinity (Bosse et al., 2012). Our analysis of nucleotide diversity outside ROH (Figure 2a) consistently resulted in greater values for Italian individuals compared to the rest of European samples which indicates that either the ancestral founder population for these breeds was large or the ancestral population received gene flow from some genetically distinct population. Both these scenarios are likely to explain these results as Italy is much closer to the centre of domestication compared to western Europe, and hence, it could be hypothesized that serial founder effect was severe in western Europe (Scheu et al., 2015) compared to Italy, while the ADMIXTURE (Figure 3a) and ancestry profile using ChromoPainter analysis (Figure 7) in this study also pointed towards presence of complex non‐European cattle ancestry in Italian cattle. Similarly, the highest π_out values (Figure 2a) observed for African taurine also indicate the possibility that the ancestral population of N'Dama (ND) was much diverse compared to European taurine. Hence, the hypothesis of introgression from diverse cattle population in the genepool of African taurine cannot be ruled out.

### 4.2 Characterizing non‐European cattle ancestry

Our results from linked and unlinked SNP‐based approaches, to detect admixture pattern, not only confirmed the previous reports but also extended the support for the complex origin of southern European cattle breeds. For instance, Decker et al. (2014) analysing 50 k SNP markers reported the presence of complex ancestry‐African taurine and zebu like‐for central Italian cattle breeds (Chianina, Romagnola and Marchigiana) and they also proposed either African or near‐eastern origin for this complex ancestry. Here, we show (Figures 3a and 7) that at least two other cattle breeds, namely Maremmana (MA) and Busha (BU), also carry similar non‐European ancestry as previously identified in Central Italian cattle. The ancestry profile of Busha, however, was slightly different from that of the Central Italian cattle. We note that it is also likely that Busha and central Italian cattle received a similar contribution from the same sources and subsequently evolve independently. The results of treemix analysis also indicate this possibility as in three out of five treemix runs, we observed Busha (BU) and Maremmana (MA) receiving migration edges from the same branch in phylogenetic networks. Nevertheless, our results (Figure 7) point towards the common origin of non‐European cattle ancestry BAI cattle breeds.

At least two hypotheses can explain the origin of the complex ancestry in BAI cattle breeds: (a) the donor populations that contributed the African and indicine ancestry in BAI cattle were different (multiple introgression events), (b) or the single donor population that carried both the ancestries contributed in the gene pool of BAI cattle breeds (single event of introgression from a single donor population). We note that it is not surprising for BAI cattle to display shared ancestry with African taurine as they both likely have the same centre of domestication. However, shared ancestry between BAI and zebu due to divergence is less likely to occur, since both the lineages are estimated to have diverged about ~250,000 YBP. To test the two hypotheses related to introgression, we applied the algorithm implemented in a tool, GLOBETROTTER (Hellenthal et al., 2014). In the absence of true admixing population, GLOBETROTTER can identify the most suitable proxy for the true donor (aka “surrogates”) among all the populations in the data set. The algorithm, however, did not identify clear admixture signal involving any surrogate population in our data set indicating that either the admixture event is very old, or the admixture events were recurring and involved multiple donor populations. Indeed, BAI cattle breeds are inhabiting in their respective regions since ancient times. Therefore, it is likely that these admixture events are too old to date using the GLOBETROTTER approach as the simulated data have shown that GLOBETROTTER can only estimate the admixture events accurately if they have occurred in about last ~170 generations (Hellenthal et al., 2014). In fact, analysing microsatellite markers from a thousand‐year‐old bone sample, Gargani et al. (2015) reported that, compared to Iberian and western European cattle, Chianina and Romagnola were genetically closer to the thousand‐year‐old ancient bovine sample. Perhaps, genotyping ancient cattle bone samples from Italy will shed more light on the age and origin of the complex ancestry present in modern central Italian cattle.

Applying three‐population tests on SNPs identified from whole‐genome sequencing data, we provide clear evidence of the cross‐bred (taurine and zebu) ancestry in an Italian Podolica sample. However, we note that this zebu ancestry might have not necessarily come from the Indian zebu itself; instead, it could have come from some zebu‐related population not sampled in our data set. Because Podolica is believed to have originated in the Podolian region of Eastern Europe, it can be hypothesized that this zebu ancestry is either of the Eastern European or western Asian origins. These results confirmed the previous hypothesis of cross‐bred origin for Podolica based on the similarity of the β‐globin variant between Podolica and Zebu (Pieragostini, Scaloni, Rullo, & Di Luccia, 2000).

Our admixture (Figure 3a) and ChromoPainter (Figure 6) results indicated shared ancestry between Iberian cattle (PA, SA and MN), Limousin (LMS) and African taurine (ND and MUT) which is in concordance with previous reports (Beja‐Pereira et al., 2003; Cymbron et al., 2005; Decker et al., 2014; Ginja, Penedo et al., 2010; Ginja, Telo Da Gama et al., 2010). Moreover, we also observed shared ancestry between EAZ and southern European cattle (Figures 3a and 6); however, as EAZ itself is a cross‐bred of African taurine and zebu, it is difficult to interpret this shared ancestry. While the clustering of LMS with Iberian cattle probably reflects the use of this commercial breed to upgrade local Iberian cattle, namely Pajuna (Martínez et al., 2012), previous studies have also reported the presence of EAZ‐specific microsatellite alleles in Iberian breeds such as Mertolenga and Pajuna (Beja‐Pereira et al., 2003; Martín‐Burriel et al., 2011). The fineStructure‐inferred tree (Figure 6) also clusters Marchigiana (MCGFH) with Chianina (CH) which is consistent with the known history of Marchigiana as a cross‐bred with a high fraction of Chianina‐related ancestry.

The three‐population tests involving British cattle as one of the reference population resulted in relatively greater significant *z*‐values for Busha and Podolica (Figure 4a,d). This could mean that British cattle is the most suitable surrogate for European taurine in our data set. We also note that the British cattle displayed the least average heterozygosity estimates (Figures 1 and 2). Taken together both these results, it is safe to propose that British cattle is the least admixed population among all European cattle populations in the data set, and thus, they might have preserved a large number of European taurine‐specific variants. Interestingly, this hypothesis may also partially explain the previous reports of British cattle sharing a high frequency of derived alleles with ancient aurochs sample, while Italian cattle reportedly displayed the least frequency of aurochs specific alleles (Park et al., 2015; Upadhyay et al., 2016).

Taken together, our results in this study provided a holistic view on non‐European cattle ancestry in southern European cattle. The fine‐scale dissection of the demographic history and evolutionary events that have shaped the genome of modern southern European cattle, however, still demand improved methods and data.

## 5 DATA ARCHIVING STATEMENTS

The whole‐genome sequencing data of 11 of the 19 newly sequenced animals are available from NCBI under the Bioproject ID PRJNA514237. The SRA id of these 11 whole‐genome sequences is SRR8426534, SRR8426535, SRR842636, SRR8426537, SRR8426538, SRR84265359, SRR8426540, SRR8426541, SRR8426542, SRR8426543, SRR8426544. The whole‐genome sequencing data of remaining 8 animals are available to interested researchers upon the request.

## CONFLICT OF INTEREST

None declared.
