# Supplementary material for: Deciphering the patterns of genetic admixture and diversity in southern European cattle using genome‐wide SNPs
Source: Evol Appl. 2019 Feb 8;12(5):951–63. doi: 10.1111/eva.12770 (PMC6503822; doi:10.1111/eva.12770)
Supplement: Supplementary file 1 [file EVA-12-951-s001.docx]

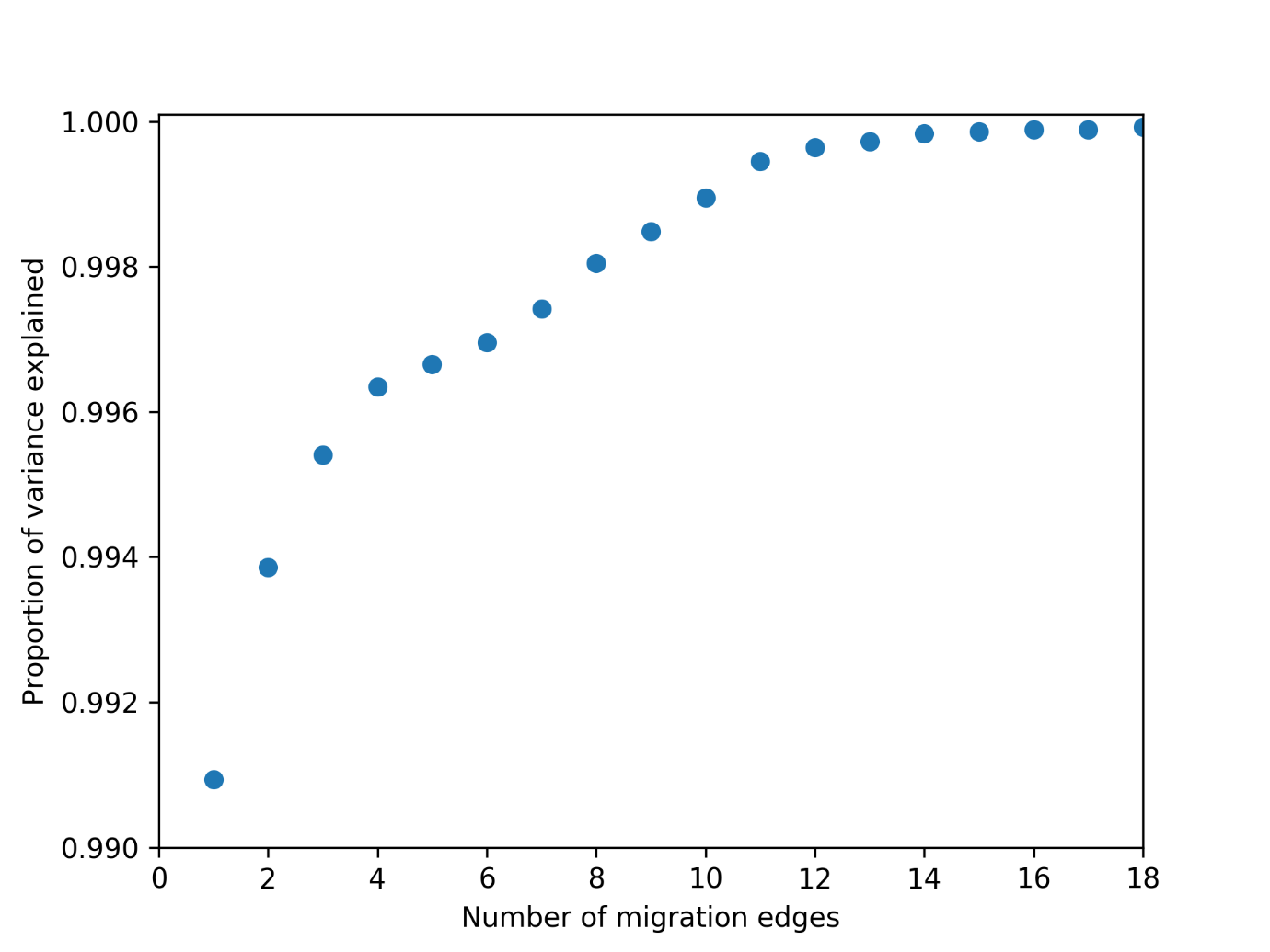


**Figure S1**. The fraction of variance in population relatedness explained by the phylogenetic models with 0 through 18 migration edges.

**Figure S2**. Two examples of phylogenetic network of inferred relationship between different cattle breeds of Europe, India and Africa.

1

2

4

EAZ

3

**Figure S3**. Clustering of individuals based on fineStructure algorithm. Note that breeds are coloured according to their geographic origin. The intensity of colour indicates shared haplotypic segments (values in terms of centiMorgan). The number beside the clusters indicate: 1. African cattle (N’Dama and East African zebu), 2. Southern European cattle (Iberian and Italian), 3. West European cattle (Commerical and British cattle).4. Zebu cattle. Note that African zebu (EAZ) clusters with Indian zebu.

**Figure S4**. Clustering of individuals based on fineStructure algorithm. Note that breeds are coloured according to their geographic origin. The intensity of colour indicates shared number of haplotypic segments based on chunklength coancestry matrix generated by ChromoPainter algorithm. The clustering pattern is exactly the same as described in Figure 6.
